# Molecular Design and Property Prediction of Sterically Confined Polyimides for Thermally Stable and Transparent Materials

**DOI:** 10.3390/polym10060630

**Published:** 2018-06-07

**Authors:** Ki-Ho Nam, Hoi Kil Choi, Hyeonuk Yeo, Nam-Ho You, Bon-Cheol Ku, Jaesang Yu

**Affiliations:** 1 Institute of Advanced Composite Materials, Korea Institute of Science and Technology (KIST), Jeonbuk 55324, Korea; khnam@kist.re.kr (K.-H.N.); 214011@kist.re.kr (H.K.C.); polymer@kist.re.kr (N.-H.Y.); 2 Department of Chemistry Education, Chemistry Building, Kyungpook National University, 80, Daehak-ro, Buk-gu, Daegu 41566, Korea; yeo@knu.ac.kr

**Keywords:** poly(ester imide), substituent effect, glass-transition temperature, molecular dynamics simulation, optical property

## Abstract

To meet the demand for next-generation flexible optoelectronic devices, it is crucial to accurately establish the chemical structure-property relationships of new optical polymer films from a theoretical point of view, prior to production. In the current study, computer-aided simulations of newly designed poly(ester imide)s (PEsIs) with various side groups (–H, –CH_3_, and –CF_3_) and substituted positions were employed to study substituent-derived steric effects on their optical and thermal properties. From calculations of the dihedral angle distribution of the model compounds, it was found that the torsion angle of the C–N imide bonds was effectively constrained by the judicious introduction of di-, tetra-, and hexa-substituted aromatic diamines with –CF_3_ groups. A high degree of fluorination of the PEsI repeating units resulted in weaker intra- and intermolecular conjugations. Their behavior was consistent with the molecular orbital energies obtained using density functional theory (DFT). In addition, various potential energy components of the PEsIs were investigated, and their role in glass-transition behavior was studied. The van der Waals energy (*E*_vdW_) played a crucial role in the segmental chain motion, which had an abrupt change near glass-transition temperature (*T*_g_). The more effective steric effect caused by –CF_3_ substituents at the 3-position of the 4-aminophenyl group significantly improved the chain rigidity, and showed high thermal stability (*T*_g_ > 731 K) when compared with the –CH_3_ substituent at the same position, by highly distorting (89.7°) the conformation of the main chain.

## 1. Introduction

In recent years, interest grew in the development of thin, lightweight, and unbreakable new-generation optoelectronic devices, with transparent flexible plastic substrates for portable devices, and roll-up and conformable displays [1,2]. At the same time, to meet the market demand for optical devices with high reliability, high integration, and rapid signal-transmission speeds, the service temperature requirements of the plastic substrates increased dramatically [3,4]. The flexible plastic substrate must be able to withstand the high temperatures of the thin-film transistor (TFT)-driven active matrix manufacturing processes, which can exceed 623 K for liquid crystal display (LCD), or active matrix organic light-emitting display (AMOLED) devices. Most common optical polymer films, such as poly(ethylene terephthalate) (PET, *T*_g_ ~ 351 K), poly(ethylene naphthalate) (PEN, *T*_g_ ~ 396 K), polycarbonate (PC, *T*_g_ ~ 423 K), and polyethersulfone (PES, *T*_g_ ~ 496 K) have limited service temperatures, and lose their optical transmittance, or deteriorate mechanically at such high manufacturing temperatures [5,6]. Temperature-resistant optical polymers therefore represent an interesting and promising class of materials because they are potential alternatives to traditional glass substrates in optoelectronic applications, with potentially better performance and durability.

Among possible candidates, wholly aromatic polyimides (PIs) have outstanding thermal stability (*T*_g_ > 523 K), are mechanically tough, and exhibit high solvent resistance and good electrical properties [7,8,9,10,11,12]. However, they have dark colors (pale yellow to brown) and poor optical transparency in optoelectronic applications [13,14]. In the aromatic PIs, this coloration and the characteristic absorption in the visible region originate from intra- and intermolecular charge transfer (CT) between the electron-accepting dianhydride and the electron-donating diamine moiety [15]. In addition, the high chain-packing order at the nanometer level of the PI degrades its optical properties due to Rayleigh scattering. Various approaches were developed to inhibit the CT interactions of conventional aromatic PIs for optoelectronic device applications, including the use of cycloaliphatic moieties, bulky pendant groups, electron-rich bridges (such as –O–), electron-withdrawing groups, and asymmetrical structures in their backbones [16,17,18,19]. However, these methods generally result in poor thermal and mechanical properties. Achieving a compromise between a light color and a high thermal stability is, therefore, a significant challenge in the development of optoelectronic polymeric substrates.

Recently, Hasegawa et al. reported various methyl- or alkyl-substituted poly(ester imide) (PEsI) systems to form colorless heat-resistant polymers [20,21]. They discussed the effect of substituents (various numbers and positions) in the ester-linked tetracarboxylic dianhydrides on the optical and thermal properties, and suggested appropriate copolymer combinations. Quite recently, we developed new aromatic PEsIs using an ester-bridged aromatic diamine with dimethyl groups at the ortho-position of the amino groups [22]. Although this PI has one of the lowest coefficients of thermal expansion (CTE) among aromatic PIs, by controlling the dihedral torsion angle between the dimethyl-substituted phenylene ring and the imide ring, it still has coloration. Thus, we continued efforts to develop PIs with high transparency, as well as high *T*_g_ and low CTE values, based on the highly twisted conformations of the phenylimides.

At the beginning phase in the development of a new optical PI polymer, it is generally necessary to investigate how numerous functional groups influence the characteristics of the PIs. This type of synthesis involves long cycles, and limitations imposed by experimental conditions. As a result, it is undesirable and expensive to attempt the synthesis of all possible PI structures without a suitable methodology of increasing the potential for success. For this reason, the ability to predict the characteristics of polymer materials from their chemical constitution is particularly important for the selection and design of new high-performance materials. Computer-aided modeling consequently became a standard method for studying complex polymer systems, and has now widely used to predict the physical properties of polymer materials. The modeling is useful for the suggestion of potential materials with predefined characteristics. For example, molecular dynamics (MD) simulation is a well-known way of studying bulk polymers at the molecular level [23,24]. In particular, it allows thermodynamic behavior, such as the glass-transition temperature (*T*_g_) and the coefficient of thermal expansion (CTE) of a candidate polymer, to be studied effectively [25,26,27,28].

Herein, we described a strategic approach to the design of transparent, high-temperature-resistant polyimides. The model compounds for the PEsIs were newly designed using different molecular structures of ester-bridged aromatic diamines, mainly bis-(4-amino-3-trifluoromethyl-phenyl) terephthalate (BATFMT), bis-(4-amino-3,5-bis-trifluoromethyl-phenyl) terephthalate (BABTFMT), and bis-(4-amino-2,3,5-tris-trifluoromethyl-phenyl) terephthalate (BATTFMT). Computer simulations were performed to predict the optical and thermomechanical properties of the PEsI model compounds. We firstly employed density functional theory (DFT) calculations to interpret the coloration of the model molecules. Details of the MD simulation were described in order to obtain insight into the molecular structure and thermal properties of the PEsIs. We obtained comparative values of the *T*_g_ and CTE of various PEsIs samples, and analyzed the role of various potential energy components in the glass-transition process. The torsion angle distributions of the PEsI chains at various temperatures were discussed to explain the chain conformations before and after the glass transition.

## 2. Methodology

### 2.1. Molecular Design

**Figure 1:**
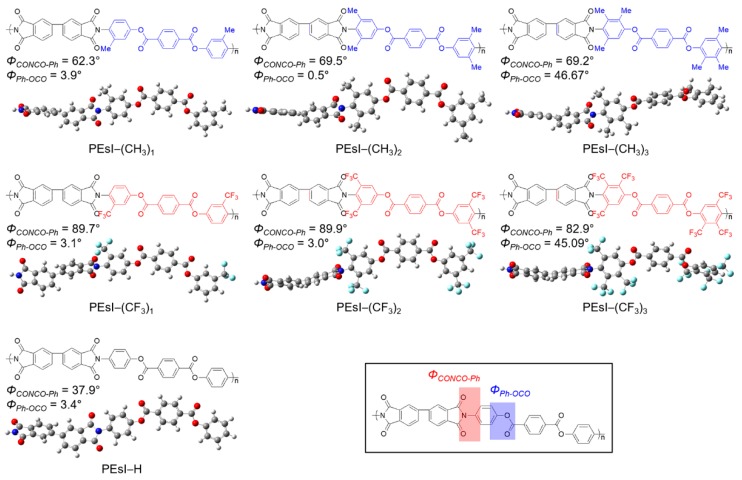
Chemical structures and three-dimensional (3D) molecular structures of the model compounds for the poly(ester imide) (PEsI) systems.

The chemical structures of the repeat units and the three-dimensional (3D) molecular structures of the model compounds for the seven PEsI models used in this study are shown in Figure 1. The seven PEsIs were synthesized from 3,3′,4,4′-biphenyltetracarboxylic dianhydride (BPDA) and corresponding diamines (i.e., bis-(4-amino-phenyl) terephthalate (BAT), bis-(4-amino-3-methyl-phenyl) terephthalate (BAMT), bis-(4-amino-3,5-dimethyl-phenyl) terephthalate (BADMT), bis-(4-amino-2,3,5,-trimethyl-phenyl) terephthalate (BATMT), bis-(4-amino-3-trifluoromethyl-phenyl) terephthalate (BATFMT), bis-(4-amino-3,5-bis-trifluoromethyl-phenyl) terephthalate (BABTFMT), and bis-(4-amino-2,3,5-tris-trifluoromethyl-phenyl) terephthalate (BATTFMT). For convenience, the PEsI models are denoted as PEsI–(CH_3_)_1_ for BPDA–BAMT, PEsI–(CH_3_)_2_ for BPDA–BADMT, PEsI–(CH_3_)_3_ for BPDA–BATMT, PEsI–(CF_3_)_1_ for BPDA–BATFMT, PEsI–(CF_3_)_2_ for BPDA–BABTFMT, and PEsI–(CF_3_)_3_ for BPDA–BATTFMT. To confirm the substituent effect, a controlled sample was prepared and labeled as PEsI–H (BPDA–BAT). To illustrate their respective possibilities, a series of PEsI–(CF)_x_ models, which were not yet synthesized, but have great potential as next-generation transparent plastic substrates, was included in this study. The PEsI–(CF)_x_ series involves modifications of the corresponding PEsI–(CH_3_)_x_ counterparts instead of –CH_3_ groups per repeat unit. Each substituent was located at the ortho- and meta-positions of the amino groups.

### 2.2. Simulation Protocol

MD simulations were performed with the Materials Studio software. In the simulations, a COMPASS II force field, including a coupling-effect energy term, was used to consider complex chain relationships in an amorphous polymer. The total potential energy equation is expressed as follows [29,30]:

(1) $$\begin{array}{cc} U_{\mathrm{total}} & =\sum_{\mathrm{stretch}}\left[k_{2}{\left(b-b_{0}\right)}^{2}+k_{3}{\left(b-b_{0}\right)}^{3}+k_{4}{\left(b-b_{0}\right)}^{4}\right]\\ & +\sum_{\mathrm{angle}}\left[k_{2}{\left(\theta -\theta_{0}\right)}^{2}+k_{3}{\left(\theta -\theta_{0}\right)}^{3}+k_{4}{\left(\theta -\theta_{0}\right)}^{4}\right]\\ & +\sum_{\mathrm{bending}}\left[k_{1}\left(1-\cos\phi \right)+k_{2}\left(1-2\cos\phi \right)+k_{3}\left(1-\cos3\phi \right)\right]+\sum_{\mathrm{torsion}}k_{2}{\left(\chi -\chi_{0}\right)}^{2}\\ & +\sum_{\mathrm{vdW}}k_{5}\left[2{\left(\frac{r_{ij}^{0}}{r_{ij}}\right)}^{9}-3{\left(\frac{r_{ij}^{0}}{r_{ij}}\right)}^{6}\right]+\sum_{\mathrm{electro}}\frac{q_{i}q_{j}}{r_{ij}}\\ & +\sum_{s,s^{\prime }}k_{6}\left(b-b_{0}\right)\left(b^{\prime }-{b^{\prime }}_{0}\right)+\sum_{s,a}k_{6}\left(b-b_{0}\right)\left(\theta -\theta_{0}\right)\\ & +\sum_{s,b}k_{6}\left(b-b_{0}\right)\left(1-\cos\phi \right)+\sum_{a,a^{\prime }}k_{6}\left(\theta -\theta_{0}\right)\left(\theta^{\prime }-{\theta^{\prime }}_{0}\right) \end{array}$$

where *U*_total_ is the total potential energy including bond balance, non-bonding, and coupling energy terms. This equation is widely used to calculate the potential energy of complex structures such as amorphous polymers. *b* is the chemical bond length, *θ* is the angle variation, *ϕ* is the bending angle, *χ* is the torsion angle, *r* is the distance between *i*th and *j*th atoms, and *q_i_* is the quantity of electric charge of *i*th atom. *k*_1_, *k*_2_, *k*_3_, *k*_4_, *k*_5_, and *k*_6_ are the force-field constants for the corresponding energy terms, and they are determined based on the type of interactive atoms. A subscript 0 indicates the value of the initial state. The cut-off distance for a non-bonding interaction was 1.50 nm.

### 2.3. Details of MD Calculations

**Figure 2:**
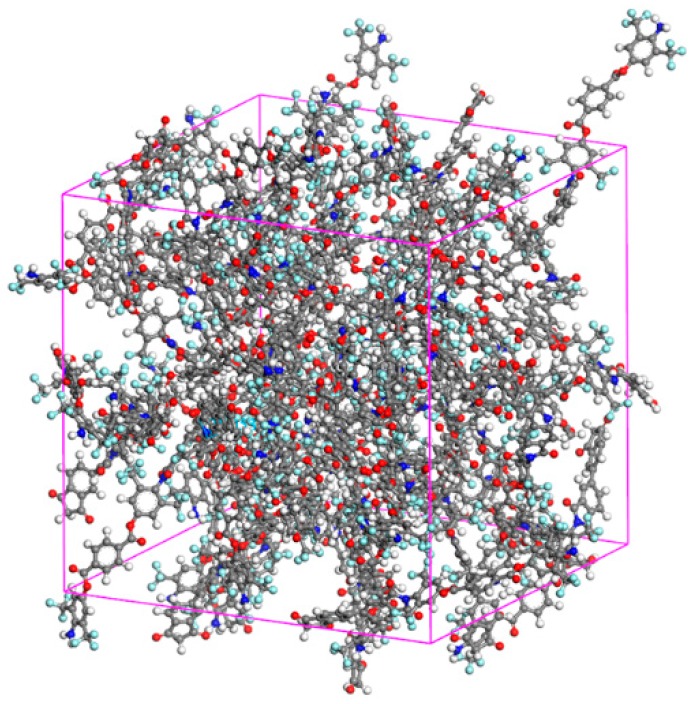
Randomly packed model compounds for PEsI–(CF_3_)_1_ in a 3D periodic boundary cell.

The PEsI chains were modeled as a unit chain (*n* = 1) to verify the interactions between the chains. The simulation cells were configured with a periodic boundary of 4.0 nm × 4.0 nm × 4.0 nm, as shown in Figure 2. The cells were packed with the PEsI unit chains with a density of 1.4 g·cm^−3^ (the density of BAT). Energy minimization of the cells was performed using a smart algorithm for the geometric optimization of the chains, and its tolerance was 10^−8^ kcal·mol^−1^. The minimized models were stabilized in isothermal and isobaric conditions (NPT ensemble). The temperature and pressure were set to room temperature (298 K) and atmospheric pressure (1 atm), respectively. This process was performed with a time step of 1 fs until the density of the simulation cell satisfied the tolerance of 10^−3^ g·cm^−3^. The average time for equilibration was about 1 ns.

The cooling-down simulations were performed to predict the relationship between the temperature and density of the simulation cell. The inner temperature of the cells was reduced from 1000 to 300 K in steps of 50 K. The NPT ensemble was performed for 500 ps at each temperature. The density for each temperature was calculated to plot the dimension change with respect to temperature. The *T*_g_ and CTE were determined from the slope and the cross point of the data fitting curves [31]. In addition, the mean-square displacements, potential energies, and torsion angle distributions of the C–N imide bonds of PEsI models were calculated to explain the influence of the substituents on the thermo-chemical characteristics of the PEsI system.

## 3. Results and Discussion

### 3.1. Molecular Orbital Calculation

**Figure 3:**
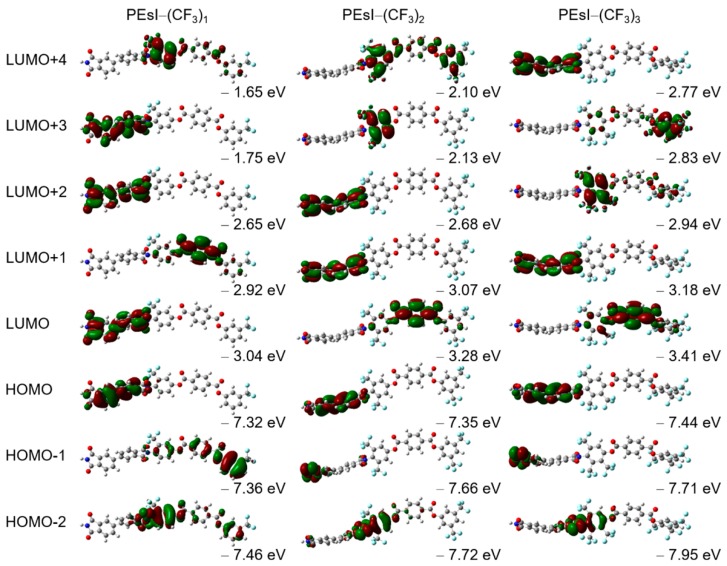
Calculated molecular orbitals and the corresponding energy levels of the repeating units of PEsI–(CF_3_)_x_ model compounds using the Becke, three-parameter, Lee-Yang-Parr (B3LYP) level with the 6-31G basis set (B3LYP/6-31G).

In order to gain insight into the coloration of the PEsIs, the software package Gaussian 09 was used for the DFT calculation [32]. Figure S1 shows a theoretical calculation of the three-dimensional (3D) molecular structures of the PEsIs, and their molecular orbital (MO) diagrams. Figure 3 provides details of the quantum-chemical calculations, molecular orbital diagrams, and calculated electronic transitions of the model compounds for the three representative systems—PEsI–(CF_3_)_1_, PEsI–(CF_3_)_2_, and PEsI–(CF_3_)_3_—at the optimized *S*_0_ geometry. The model compounds were composed of the basic units of the polymers, and their electronic transitions were calculated using the Becke, three-parameter, Lee-Yang-Parr (B3LYP) level with the 6-31G basis set [32]. Irrespective of the nature of the tetracarboxylic dianhydride, the calculated dihedral angles of a series of PEsI–(CF_3_)_x_ between the –CF_3_-substituted phenylene ring and the imide ring at round states were in the range of 82.9–89.9°, which was significantly larger than the dihedral angles observed in the unsubstituted PEsI–H and –CH_3_-substituted PEsI–(CH_3_)_x_ system. Note that the steric hindrance, caused by the bulky –CF_3_ groups directly attached to the aromatic BAT, forced the imide bond to be almost perpendicular, thereby causing significant conformational distortion of the PEsI backbones. The greater dihedral angle of the PEsI–(CF_3_)_x_ model was caused by the steric hindrance of the –CF_3_ groups, which reduced the close chain packing, resulting in a more colorless and transparent nature. Furthermore, the more twisted structure of the PEsI–(CF_3_)_x_ model resulted in largely isolated electronic states of the molecular orbitals. The number of –CF_3_ groups had a greater influence on the optical properties than their positions in the PEsI backbone. The results showed that the judicious introduction of electron-withdrawing –CF_3_ groups to the diamine moiety of PEsIs, which had considerable steric hindrance on the backbone, was quite effective at eliminating or at least reducing the characteristic coloration of the aromatic PEsIs.

### 3.2. Determination of the T_g_ and CTE

**Figure 4:**
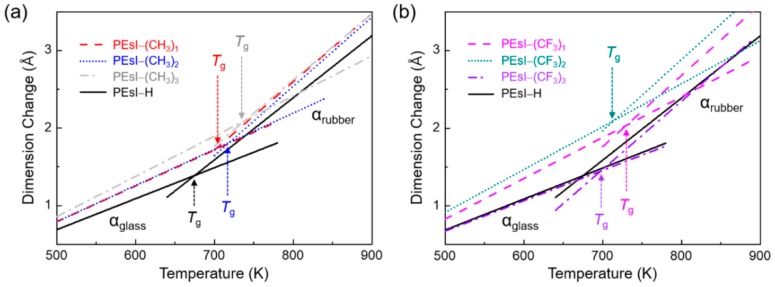
Plots of dimension change versus temperature obtained from molecular dynamics (MD) simulations. (**a**) –CH_3_- and (**b**) –CF_3_-substituted PEsI systems.

One of the most important parameters for the fabrication of the optical device is the *T*_g_. The temperature dependence of the dimension change of the PEsI models is shown in Figure 4. Dimension change means the linear change of cell length in the MD simulation. The dimension change exhibited a linear relationship with temperature at temperatures lower (*α*_glass_, glassy region) or higher (*α*_rubber_, rubbery region) than the *T*_g_. Therefore, the *T*_g_ was determined by the point of intersection of the slopes corresponding to the heating and cooling processes. The *T*_g_s of the seven PEsIs were confirmed from the plots of dimension changes, obtained from the NPT dynamics versus temperature, for temperatures ranging below and above *T*_g_. The *T*_g_ values of the PEsI–(CH_3_)_x_ series ranged from 702 to 737 K, and the PEsI–(CF_3_)_x_ series ranged from 697 to 731 K, much higher than that of the PEsI–H’s (677 K). It was found that the *T*_g_ values of the PEsIs were related to the structural effects of the aromatic diamines. The PEsIs having a –CH_3_ substituent exhibited a higher *T*_g_ in the order of PEsI–(CH_3_)_3_ > PEsI–(CH_3_)_2_ > PEsI–(CH_3_)_1_. The results showed that the incorporation of –CH_3_ groups at the ortho- and meta-positions of the amino groups effectively increased *T*_g_ values, by increasing the chain rigidity of the PEsI; that is, the –CH_3_ groups significantly constrained bond rotation around the C–N imide bonds. The PEsI model was quite effective at increasing the *T*_g_ when the –CF_3_ substitution was introduced at the 3-position of the 4-aminophenyl moiety. This was supported by the fact that the highly twisted dihedral angles of the C–N imide bonds in the optimized geometry (Figure 1) of the models were 89.7°, 89.9°, and 82.9° for PEsI–(CF_3_)_1_, PEsI–(CF_3_)_2_, and PEsI–(CF_3_)_3_, respectively. The bulkier –CF_3_ substitution stiffened the polymer main chain more effectively than the –CH_3_ unit by introducing a barrier to segmental rotation. Despite the significant increase in *T*_g_ in PEsI–(CF_3_)_1_ when compared with its analogous counterpart (PEsI–(CH_3_)_1_), the *T*_g_ values in PEsI–(CF_3_)_2_ and PEsI–(CF_3_)_3_ were found to be slightly reduced. This could be explained by the disturbed inter-chain interactions from the high free volume present in PEsI–(CF_3_)_x_, owing to their bulky –CF_3_ moieties and non-coplanar structure.

The CTE was determined to be the proper parameter for the estimation of the agreement between the theoretical results and experimental data because accurately determining *T*_g_ using MD simulations is rather complicated. The CTE was established using the relationship of temperature change and deformation. The dimension change with respect to temperature was obtained during the abovementioned cooling-down simulations. The CTE (*α*) was expressed as follows:

(2) $$\alpha =\frac{S}{\Delta T}\varepsilon_{T}=\frac{S}{\Delta T}\left(\frac{L}{L_{0}}-1\right)=\frac{S}{\Delta T}\left(\frac{\sqrt[{3}]{V}}{\sqrt[{3}]{V_{0}}}-1\right),$$

where *α* is the linear thermal expansion coefficient, *L* and *V* are the length and volume of the simulation cell, respectively, Δ*T* is temperature change, and subscript 0 indicates the initial state. *S* is a scale factor (=10^−6^) to convert the units of CTE to ppm. The CTE was calculated from the slope of the curve of dimension change versus temperature, and the initial simulation-cell length.

Table 1 shows the calculated CTEs for the PEsIs in the glassy and rubbery states compared with the experimental values available from our previous work. It can be seen that the computed values for the CTEs of the PEsI–H and PEsI–(CH_3_)_2_ were in very good agreement with the experimental data (Figure S2). All of the pseudo rigid-rod-like structures of the BPDA-based PEsI model molecules had low CTE values of less than 14 ppm·K^−1^ in the MD calculations. Considering that the CTE of current glass substrates is 5–9 ppm·K^−1^ [33], the CTE values of the PEsIs were within the effective range for applications as a plastic substrate. Interestingly, despite the bulky –CF_3_ groups interfering with chain packing, the PEsI–(CF_3_)_x_ series showed a low level of CTEs similar to the PEsI–(CH_3_)_x_ series. This suggested that the considerably distorted conformations in the PEsI systems effectively constrained the degree of rotational freedom at the C–N imide bonds, leading to the strong chain stiffness of the macromolecules. The steric effect was more noticeable for the PEsI–(CF_3_)_3_ because they had two different types of steric hindrance: between the imide ring and the benzene ring, and between the two benzene rings of the diamine along the ester-linkage axis. Therefore, the consecutive rigid rings along the main chains in the PEsI–(CF_3_)_3_ had significantly distorted conformations. These results provided valuable information about the thermal properties of the newly designed polymers, which were previously limited to empirical measurements.

### 3.3. Mean-Square Displacement

*T*_g_ was well correlated with the rigidity of the polymer, specifically the segmental motion in the polymer chain. To observe the mobility of the PEsI chains during the glassy to rubbery phase transition, the mean-square displacements (MSDs) of the PEsI molecular systems were calculated [31]. The MSD of *N* atoms is expressed as follows:

(3) $$\mathrm{MSD}=\frac{1}{3N}\sum_{i=0}^{N-1}\langle {\left|{\vec{R}}_{i}\left(t\right)-{\vec{R}}_{i}\left(0\right)\right|}^{2}\rangle ,$$

where *R_i_*(*t*) indicates the position vector of the *i*th atom at time *t* [34]. The MSD curves for each temperature were obtained from the values of the initial 50 ps of each NPT ensemble. The relative dispersion of polymer chains was estimated from the variation in the MSD curve with temperature. When the temperature of the simulation cell reached *T*_g_, its relative dispersion suddenly increased. Thus, the temperature ranges between the points where the MSD curves suddenly increased were candidates for the glass-transition region. The glassy and rubbery regions were classified using these temperature ranges, and each region had independent characteristics. Therefore, by investigating the interval between each MSD curve, an approximate candidate range for the *T*_g_ was estimated within 50 K.

**Figure 5:**
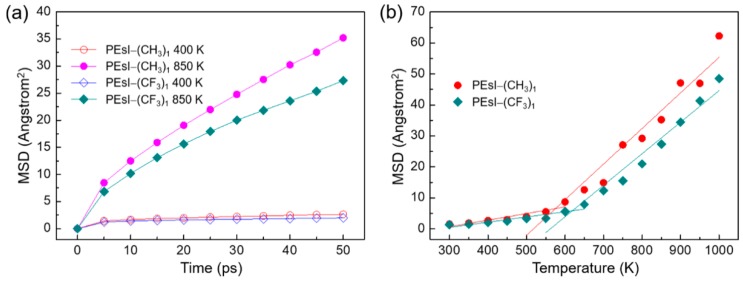
(**a**) Mean-square displacement (MSD) curves as a function of time for PEsI–(CH_3_)_1_ and PEsI–(CF_3_)_1_ at 400 and 850 K. (**b**) MSD curves as a function of temperature at 50-ps time intervals for the unit cells of the PEsI–(CH_3_)_1_ and PEsI–(CF_3_)_1_ systems.

As shown in Figure S3, the motion of the chain segment of the PEsI systems increased consistently with the increasing temperature. As an example, using PEsI–(CH_3_)_1_ and PEsI–(CF_3_)_1_, the MSD plots for the two PEsI systems at 400 and 850 K are illustrated in Figure 5a. It shows that the MSD variation between 400 and 800 K for the PEsI–(CF_3_)_1_ system was lower than that for the PEsI–(CH_3_)_1_ system’s all-time range. The bulky –CF_3_ groups were generally believed to form a loosely packed PI structure, but their bulky structure effectively induced a highly twisted form in phenylimide due to the structural steric effect of the substituents. This caused the ortho –CF_3_ substituent of PEsI–(CF_3_)_1_ to stiffen the polymer main chain more effectively, by providing a barrier to segmental rotation. Hence, it needed more time to produce the segmental motion of PEsI–(CH_3_)_1_ than its counterparts. The slope of the MSD above the *T*_g_ value was much higher than that below *T*_g_, indicating that each of the PEsI chains had higher mobility above the glass-transition region. This abrupt change between the two temperature zones, based on the *T*_g_, can be seen more clearly in Figure 5b, where the MSD is plotted as a function of temperature at 50-ps time periods. The *T*_g_ of the PEsIs was also identified through the slope change of the MSD curve of the chain segments versus the temperature [35]. It can be seen that the *T*_g_s of all PEsI systems (Figure S4) were above 400 K, and consistent with the results described in Section 3.2.

### 3.4. Role of the Energy Components

**Figure 6:**
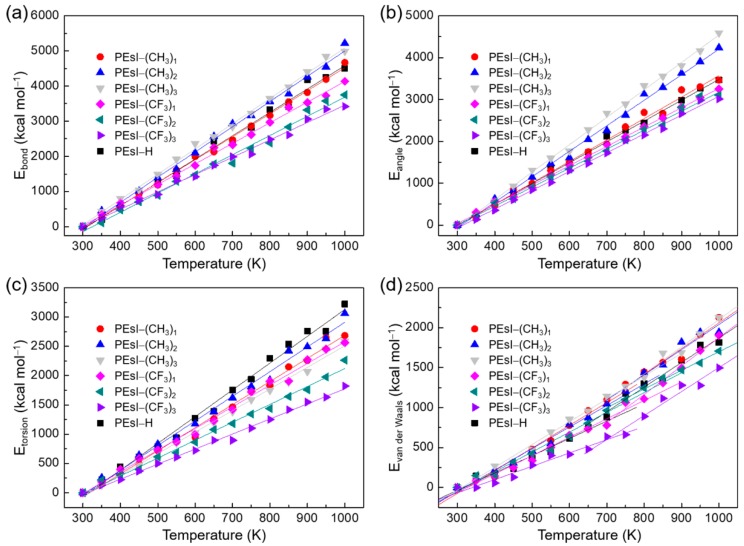
Plots of potential energy components versus temperature of the PEsI systems. (**a**) Bond stretching energy (*E*_bond_), (**b**) angle variation energy (*E*_angle_), (**c**) dihedral torsional energy (*E*_torsion_), and (**d**) van der Waals energy (*E*_vdW_).

Various interaction-energy components were also used to investigate the glass transition occurring in the PEsIs [36,37,38]. The calculated results of bond stretching energy (*E*_bond_), angle variation energy (*E*_angle_), dihedral torsional energy (*E*_torsion_), and van der Waals energy (*E*_vdW_) versus the temperature are plotted in Figure 6. In the *E*_vdW_ versus temperature plot, it can be seen that there was a break indicating where the glass transition occurred. In the regions both below *T*_g_ and above *T*_g_, *E*_vdW_ had a linear relationship with temperature, with a break at *T*_g_. However, the other potential energy components continued to increase linearly with increasing temperature without any break. These results indicated that the *E*_vdW_ played a crucial role in the glass-transition process of the PEsI systems.

### 3.5. Torsion Angle Distribution

**Figure 7:**
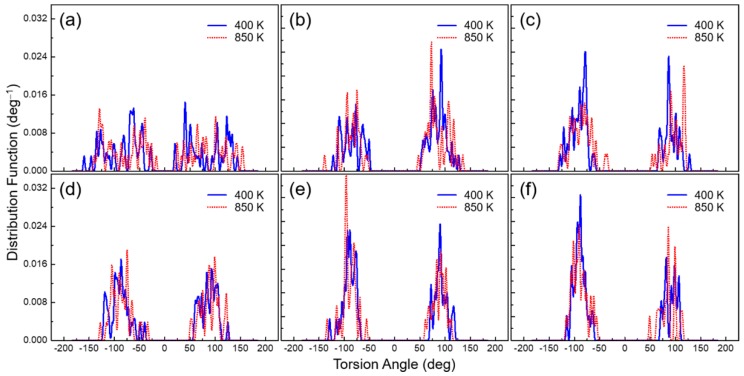
Torsion angle distributions of the PEsI systems obtained from the MD simulations at 400 and 850 K. (**a**) PEsI–(CH_3_)_1_; (**b**) PEsI–(CH_3_)_2_; (**c**) PEsI–(CH_3_)_3_; (**d**) PEsI–(CF_3_)_1_; (**e**) PEsI–(CF_3_)_2_; and (**f**) PEsI–(CF_3_)_3_.

The mobility of the C–N imide bonds, provided by the substituent patterns, exerted a significant influence on the glass-transition process. The investigated torsion (*Φ_CONCO-Ph_*) along the chemical backbone of the substituted PEsIs was set from –180° to +180°, and is defined in Figure 7. The PEsIs had a different peak position and relative proportion for *Φ_CONCO-Ph_* (the torsion angle between the imide and phenyl rings) depending on their molecular structures. PEsI–H (Figure S5) and PEsI–(CH_3_)_1_ had many peaks over the range of ±15° to ±165°, but there were only two major peaks located in the ±90° range in other PEsI systems. These were attributable to the ortho- and/or meta-substitution in the aromatic diamine compounds, which induced significant steric hindrance around the C–N imide bonds. Notably, the steric hindrance between adjacent groups, derived from the 3-trifluoromethyl moiety, greatly restricted the torsional bond rotation of the C–N imide bonds. Actually, the probability distribution of the torsion angle at *Φ_CONCO-Ph_* with temperature was narrower in the PEsI–(CF_3_)_x_ series. This result was consistent with the torsion angles obtained in the molecular structures of the stabilized model compounds (Figure 1). To investigate the effect of temperature on torsion-angle distribution, the torsion-angle distributions of the PEsI series were obtained at 400 and 850 K. These distribution functions showed peaks at the same position for the two temperatures, and had a wider distribution at higher temperatures; however, they showed a slight difference in probability densities. Regardless of the glassy or rubbery state, the torsion angle of the PEsIs, excluding PEsI–H and PEsI–(CH_3_)_1_, remained at approximately ±90°. This proved that the *E*_torsion_ was not abruptly changed by the glass-transition process, due to the limited torsion discussed in Section 3.3. In conclusion, *T*_g_ was found to correlate with the rotation-barrier height of the imide bond.

Table S1 shows a final comparison of the *T*_g_ and CTE values of PEsI–(CF_3_)_1_ with fluorinated PIs, taken from previous work in the literature. From these data, it became clear that the current methodology of formation of the highly distorted conformation at the C–N imide bonds resulted in high-temperature-resistant (*T*_g_ > 731 K) and highly transparent optical polymers. We also believed that the introduction of a small number of –CF_3_ groups at only the ortho-positions of the amino groups could potentially reduce the production cost of fluorinated PIs, for applications in the optoelectronic field.

## 4. Conclusions

In this study, we designed seven polymer models that have the potential of being future optical polymer films, and computed their thermomechanical and optical properties. The thermal behavior of the PEsIs was investigated using various MD calculations. The *T*_g_ of polymeric materials was mainly determined by the chain rigidity (time scale) and the strength of the inter-chain interactions (space scale). The theoretically found *T*_g_s of the PEsI systems consistently reproduced the chemical structure-property relationships in polymer backbone chain rigidity, and the substituent effects on the glass-transition process. Potential energy components, such as *E*_vdW_ and *E*_torsion_, played significant roles in the glass-transition behavior of the PEsIs, as shown by the plots of the energy components against temperatures ranging from below *T*_g_ to above *T*_g_. The –CF_3_ substituent in the PEsI backbone structure reduced the intra- and intermolecular charge-transfer complexes, keeping their rigid-rod-like backbones intact. It is very inspiring that, by applying MD simulations and DFT calculations, we found a new polymer model (PEsI–(CF_3_)_1_) that satisfied both high optical and thermomechanical properties. This work provides a useful approach to the design of transparent, highly temperature-resistant molecular systems for advanced optical applications, when compared with the present fluorine chemistry of PIs.

## Figures and Tables

**Table 1:** Glass-transition temperature (*T*_g_) and thermal expansion coefficients (*α*) of the seven poly(ester imide) (PEsI) models.

Sample code	*T*_g_^exp^ (K)	*T*_g_ (K)	*α*^exp^ (ppm·K^−1^)	*α*^glassy^ (ppm·K^−1^)	*α*^rubbery^ (ppm·K^−1^)
PEsI–(CH_3_)_1_	–	701.5	NA	11.2	21.4
PEsI–(CH_3_)_2_	>623	720.8	11.3	11.3	21.7
PEsI–(CH_3_)_3_	–	737.4	–	12.6	20.8
PEsI–(CF_3_)_1_	–	730.9	–	12.8	22.3
PEsI–(CF_3_)_2_	–	714.9	–	13.4	22.3
PEsI–(CF_3_)_3_	–	696.9	–	9.5	21.3
PEsI–H	>623	676.9	8.2	9.7	19.5

The data of experimental *T*_g_ (PEsI–Me_2_ and PEsI–H) cited in this table were investigated using dynamic mechanical thermal analysis (DMA) at a heating rate of 3 °C·min^−1^ with a load frequency of 1 Hz in air, and were reproduced from Reference [22] with permission from ELSEVIER, Copyright 2017.

## Supplementary Materials

The following are available online at http://www.mdpi.com/2073-4360/10/6/630/s1: Figure S1: Calculated molecular orbitals and the corresponding energy levels of the basic units of PEsI–H and PEsI–(CH_3_)_x_ model compounds (B3LYP/6–31G); Figure S2: DMA thermograms of the PEsI–H and PEsI–(CH_3_)_2_ films (1 Hz, 3 °C·min^−1^), (a) storage modulus and (b) tan δ; Figure S3: MSD of the structures as a function of time for the PEsI systems, (a) PEI–(CH_3_)_1_, (b) PEI–(CH_3_)_2_, (c) PEI–(CH_3_)_3_, (d) PEI–(CF_3_)_1_, (e) PEI–(CF_3_)_2_, and (f) PEI–(CF_3_)_3_; Figure S4: MSD curves as a function of temperature at 50-ps time intervals for the unit cells of PEsI systems; Figure S5: Torsion angle distributions of the PEsI–H systems derived from NVT MD simulations at 400 and 850 K; and Table S1: Thermal properties of fluorinated PIs.
