# Supplementary material for: Molecular Design and Property Prediction of Sterically Confined Polyimides for Thermally Stable and Transparent Materials
Source: Polymers (Basel). 2018 Jun 7;10(6):630. doi: 10.3390/polym10060630 (PMC6403577; doi:10.3390/polym10060630)
Supplement: Supplementary file 1 [file polymers-10-00630-s001.pdf]

Supporting Information

Molecular Design and Property Prediction of Sterically Confined Polyimides for Thermally Stable and Transparent Materials

Ki-Ho Nam [1,†], Hoi Kil Choi [1,†], Hyeonuk Yeo [2], Nam-Ho You [1], Bon-Cheol Ku [1,*], and Jaesang Yu [1,*]

[1] *Institute of Advanced Composite Materials, Korea Institute of Science and Technology (KIST), Jeonbuk 565-902, Republic of Korea*

[2] *Department of Chemistry Education, Chemistry Building, Kyungpook National University, Chemistry Building, 80, Daehak-ro, Buk-gu, Daegu 41566, Republic of Korea*

Author Information

* Corresponding authors.

† These authors contributed equally.

List of Contents for Supplementary Material:

Figure S1. Calculated molecular orbitals and the corresponding energy levels of the basic units of PEsI–H and PEsI–$(CH_3)_x$ model compounds (B3LYP/6–31G).

Figure S2. DMA thermograms of the PEsI–H and PEsI–$(CH_3)_2$ films (1 Hz, 3 °C min^{-1}). (a) storage modulus and (b) tan δ.

Figure S3. MSD of the structures as a function of time for the PEI systems. (a) PEI–$(CH_3)_1$, (b) PEI–$(CH_3)_2$, (c) PEI–$(CH_3)_3$, (d) PEI–$(CF_3)_1$, (e) PEI–$(CF_3)_2$, and (f) PEI–$(CF_3)_3$.

Figure S4. MSD curves as a function of temperature at 50 ps long time for the unit cell of PEsI systems.

Figure S5. Torsion angle distributions of the PEsI–H systems by constant-NVT MD simulation at 400 and 850 K.

Table S1. Thermal properties of fluorinated PIs

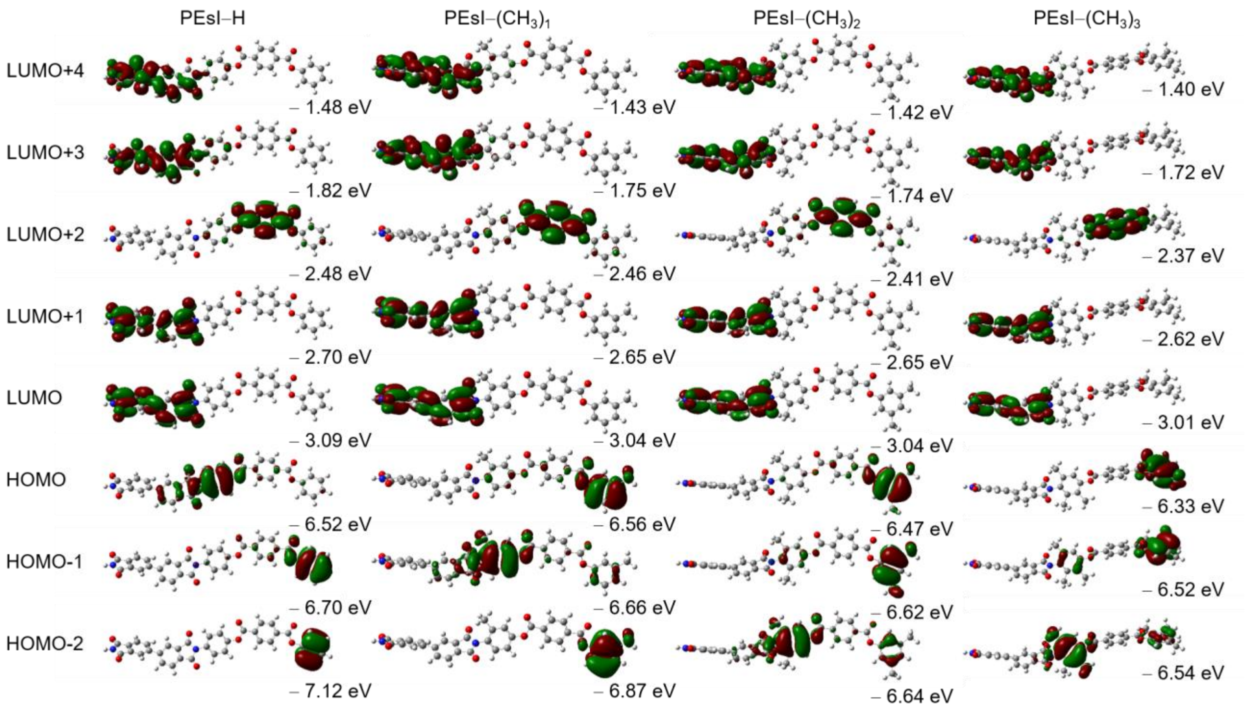

Figure S1. Calculated molecular orbitals and the corresponding energy levels of the basic units of PEsI–H and PEsI–$(CH_3)_x$ model compounds (B3LYP/6–31G).

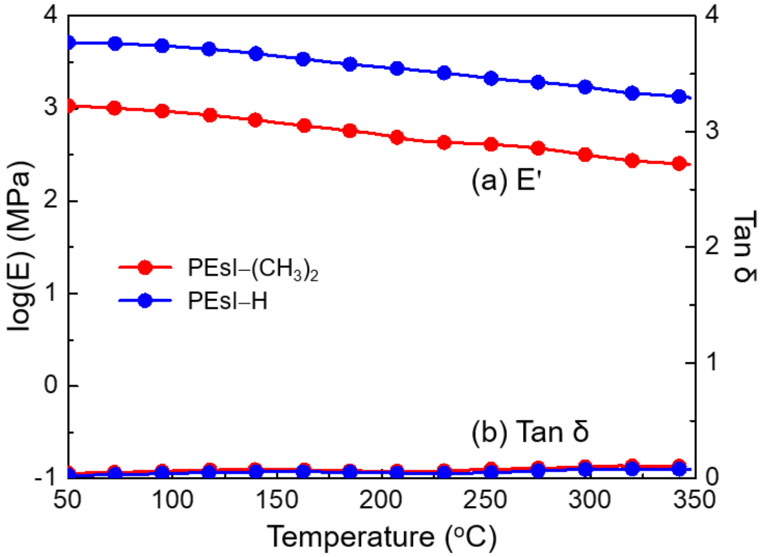

Figure S2. DMA thermograms of the PEsI–H and PEsI–$(CH_3)_2$ films (1 Hz, 3 °C min^{-1}). (a) storage modulus and (b) tan δ.

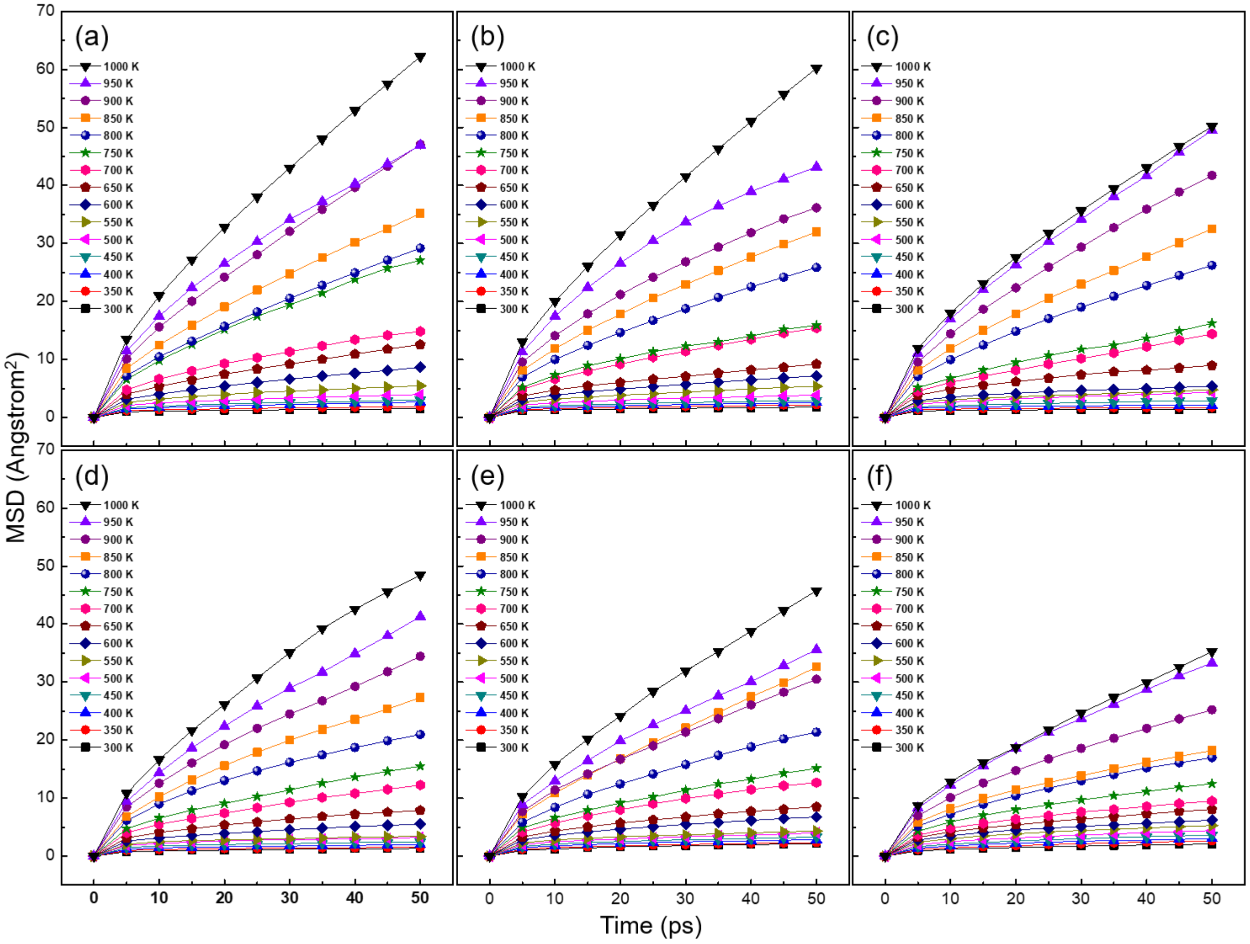

Figure S3. MSD of the structures as a function of time for the PEI systems. (a) PEI–$(CH_3)_1$, (b) PEI–$(CH_3)_2$, (c) PEI–$(CH_3)_3$, (d) PEI–$(CF_3)_1$, (e) PEI–$(CF_3)_2$, and (f) PEI–$(CF_3)_3$.

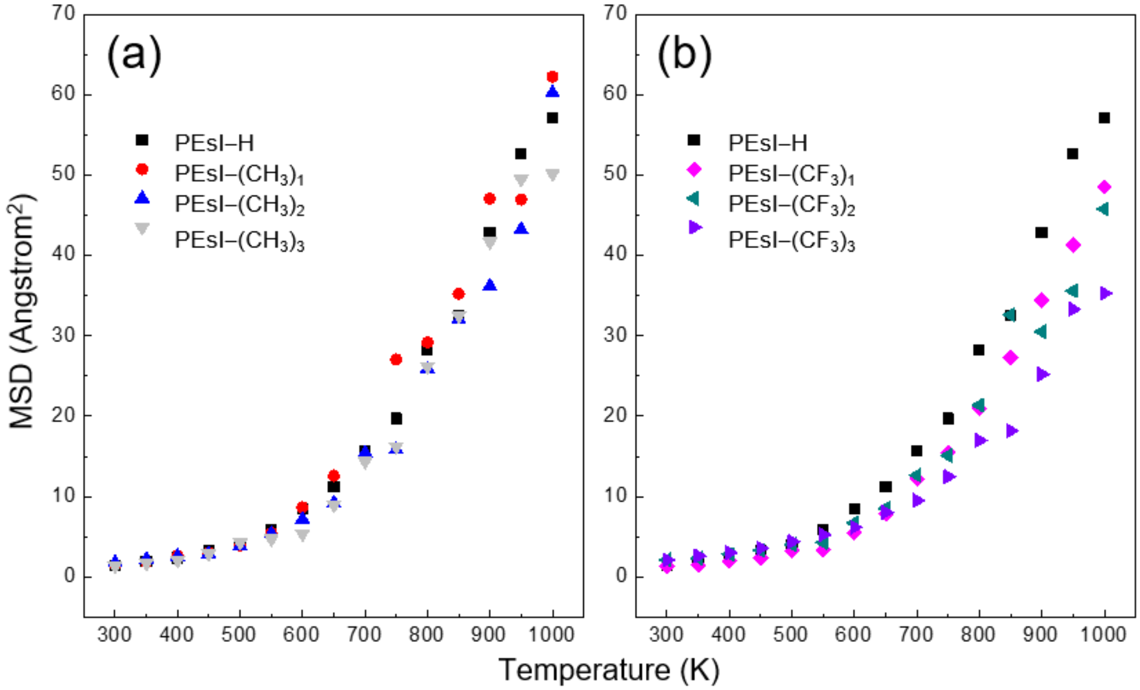

Figure S4. MSD curves as a function of temperature at 50 ps long time for the unit cell of PEsI systems.

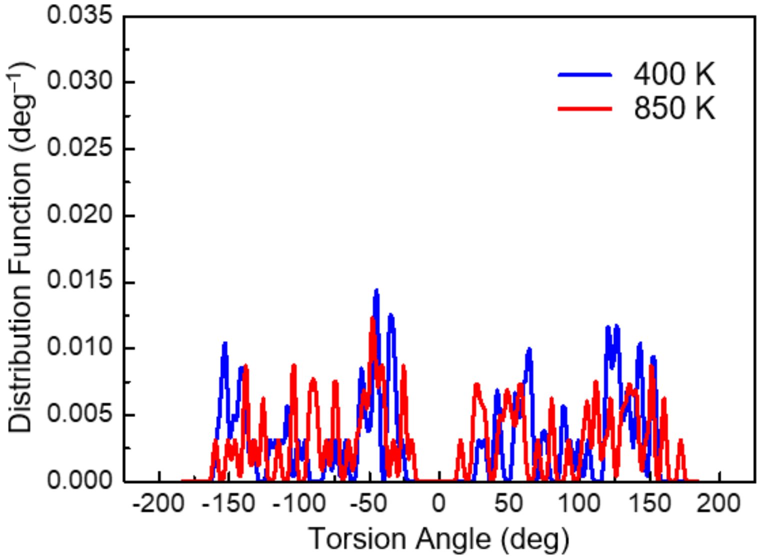

Figure S5. Torsion angle distributions of the PEsI–H systems by constant-NVT MD simulation at 400 and 850 K.

Table S1. Thermal properties of fluorinated PIs

PI type	Molecular structure	T_g (K)	CTE (ppm K^{-1})	Reference
Fluorinated ester-bridged PI	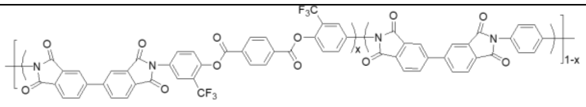 x = 5	651	8.3	Yang, S. et al.[1]
	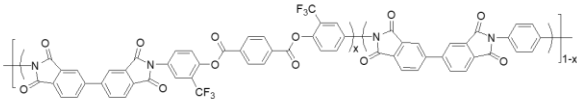 x = 30	587	18.3	
PI containing pyridine and fluorinated units	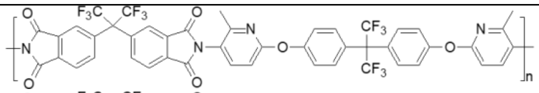	535	68	Shang, D. et al.[2]
	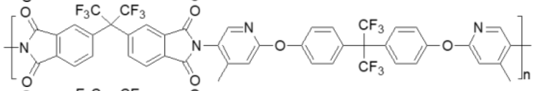	548	64	
	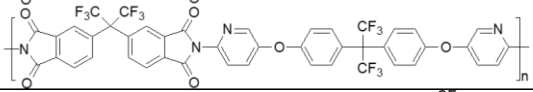	538	67	
PI derived from fluorinated tetracarboxylic dianhydride	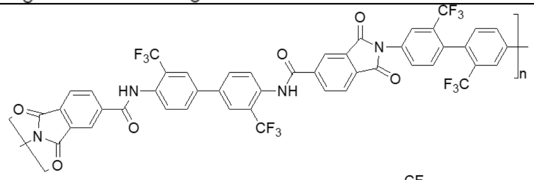	543	8.3	Hasegawa, M. et al.[3]
	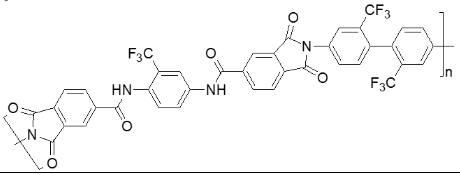	505	26.5	
PI containing ether and fluorinated units	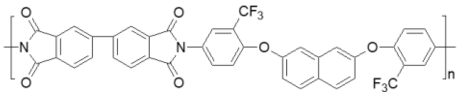	544	NA	Hsiao, S. et al.[4]
PI derived from multi-trifluoromethyl-substituted aromatic diamine	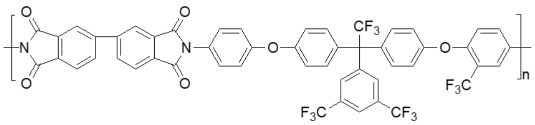	512	62.1	Yang, S. et al.[5]
Our model compounds	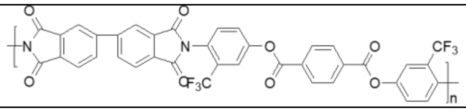	731	12.8	This work

References

1. Chen, W.; Liu, F.; Ji, M.; Yang, S. Synthesis and characterization of low-CTE polyimide films containing trifluoromethyl groups with water-repellant characteristics. *High Perform. Polym.* **2017**, *29*, 501–512.

2. Dong, W.; Guan, Y.; Shang, D. Novel soluble polyimides containing pyridine and fluorinated units: Preparation, characterization, and optical and dielectric properties. *RSC Adv.* **2016**, *6*, 21662–21671.

3. Hasegawa, M.; Ishigami, T.; Ishii, J.; Sugiura, K.; Fujii, M. Solution-processable transparent polyimides with low coefficients of thermal expansion and self-orientation behavior induced by solution casting. *Eur, Polym. J.* **2013**, *49*, 3657–3672.

4. Hsiao, S.-H.; Yang, C.-P.; Chung, C.-L. Synthesis and characterization of novel fluorinated polyimides based on 2,7-bis(4-amino-2-trifluoromethylphenoxy)naphthalene. *J. Polym. Sci. Pol. Chem.* **2003**, *41*, 2001–2018.

5. Tao, L.; Yang, H.; Liu, J.; Fan, L.; Yang, S. Synthesis and characterization of highly optical transparent and low dielectric constant fluorinated polyimides. *Polymer* **2009**, *50*, 6009–6018.
